# Supplementary material for: Homophilic and heterophilic cadherin bond rupture forces in homo- or hetero-cellular systems measured by AFM-based single-cell force spectroscopy
Source: Eur Biophys J. 2021 Apr 20;50(3-4):543–59. doi: 10.1007/s00249-021-01536-2 (PMC8190030; doi:10.1007/s00249-021-01536-2)
Supplement: Supplementary file 1 — Supplementary file1 (DOCX 3494 KB) [file 249_2021_1536_MOESM1_ESM.docx]

Homophilic and heterophilic cadherin bond rupture forces in homo- or hetero-cellular systems measured by AFM based SCFS

Prem Kumar Viji Babu^1^, Ursula Mirastschijski^2^, Gazanfer Belge^3^, Manfred Radmacher^1*^

^1^Institute of Biophysics, University of Bremen, Bremen, Germany

^2^Wound Repair Unit, Centre for Biomolecular Interactions Bremen, University of Bremen, Bremen, Germany

^3^Faculty of Biology and Chemistry, University of Bremen, Bremen, Germany

*To whom correspondence should be addressed. E-mail: mr@biophysik.uni-bremen.de

**Supplementary Information**

**
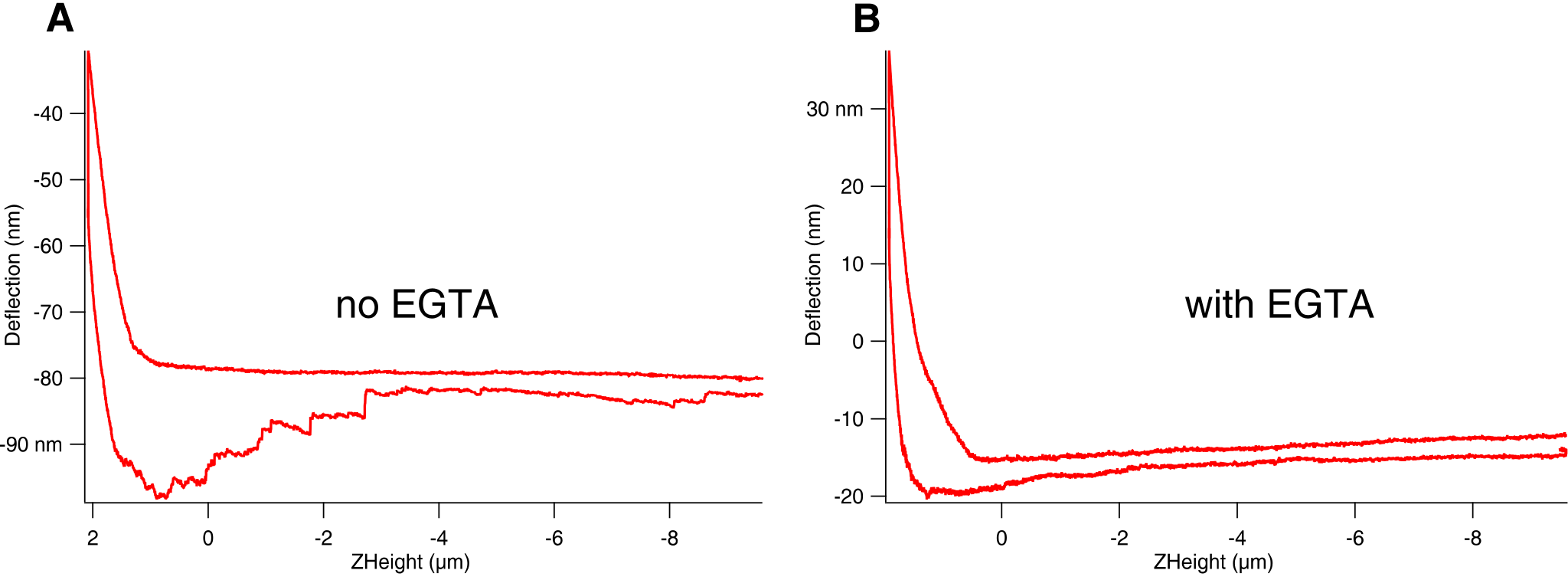
**

**Supplementary figure 1** Force curves obtained during cell-cell interaction (here MDCK-MDCK) show distinct rupture events under normal conditions (no EGTA) (A), which in the presence of EGTA, corresponding to low or no Ca^2+^ present, disappear (B).

**
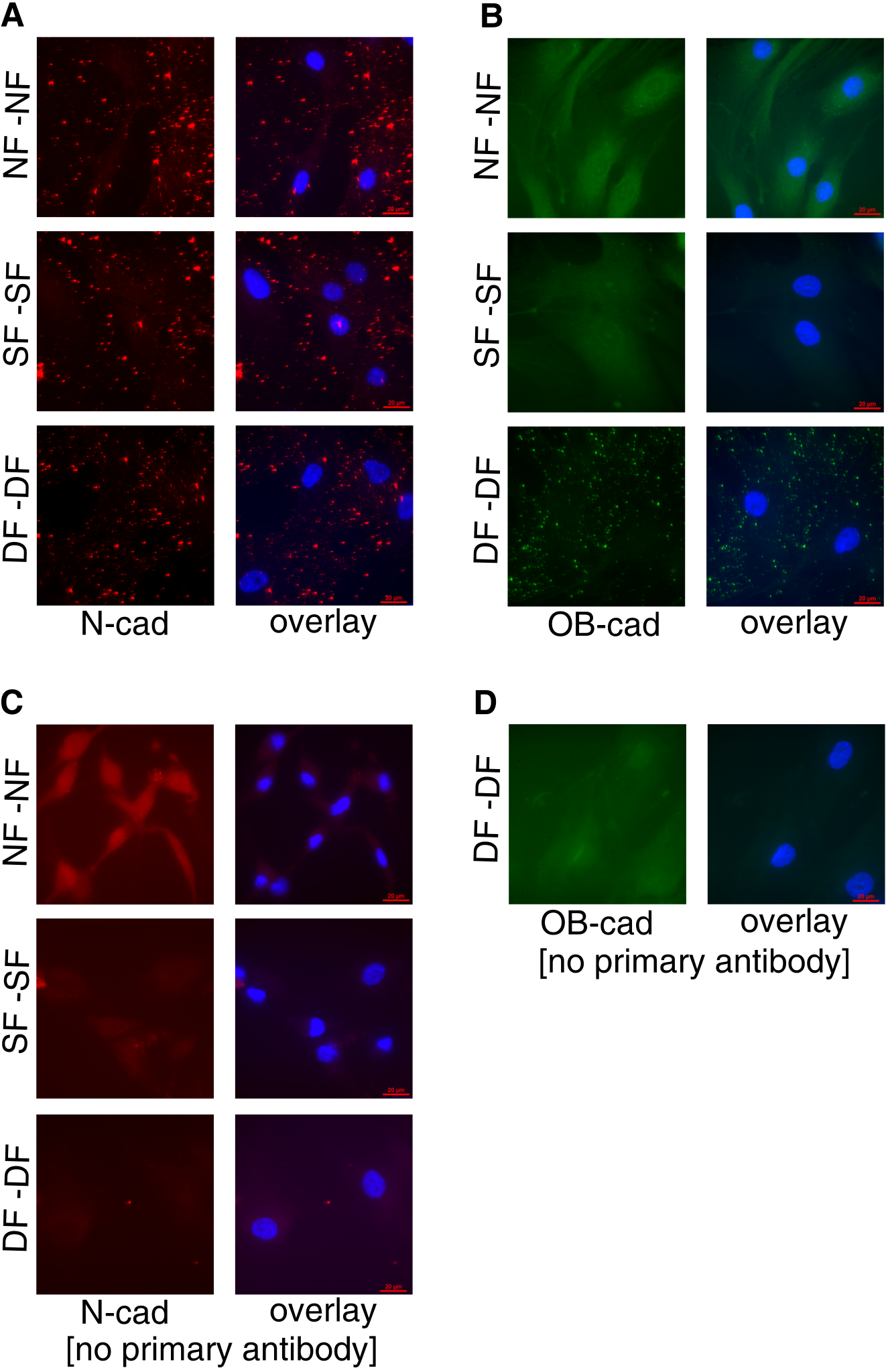
**

**Supplementary figure 2** Immunostaining of fibroblasts adherens junctions for N- and OB- cadherin shows N-cad expression (red fluorescence) in all fibroblasts (A) and OB-cad expression (green fluorescence) only in DF interaction sites (B). In the right column (overlay) DAPI staining of the nucleus is overlayed with the corresponding antibody staining. In the control measurements (C&D) unspecific binding of the secondary antibody was checked by staining without the corresponding primary antibody for N-cad (C) and OB-cad (D). Only a weak homogenous background fluorescence signal was detected showing that the secondary antibody specifically binds the primary antibody. Scale bar 20 µm.

**
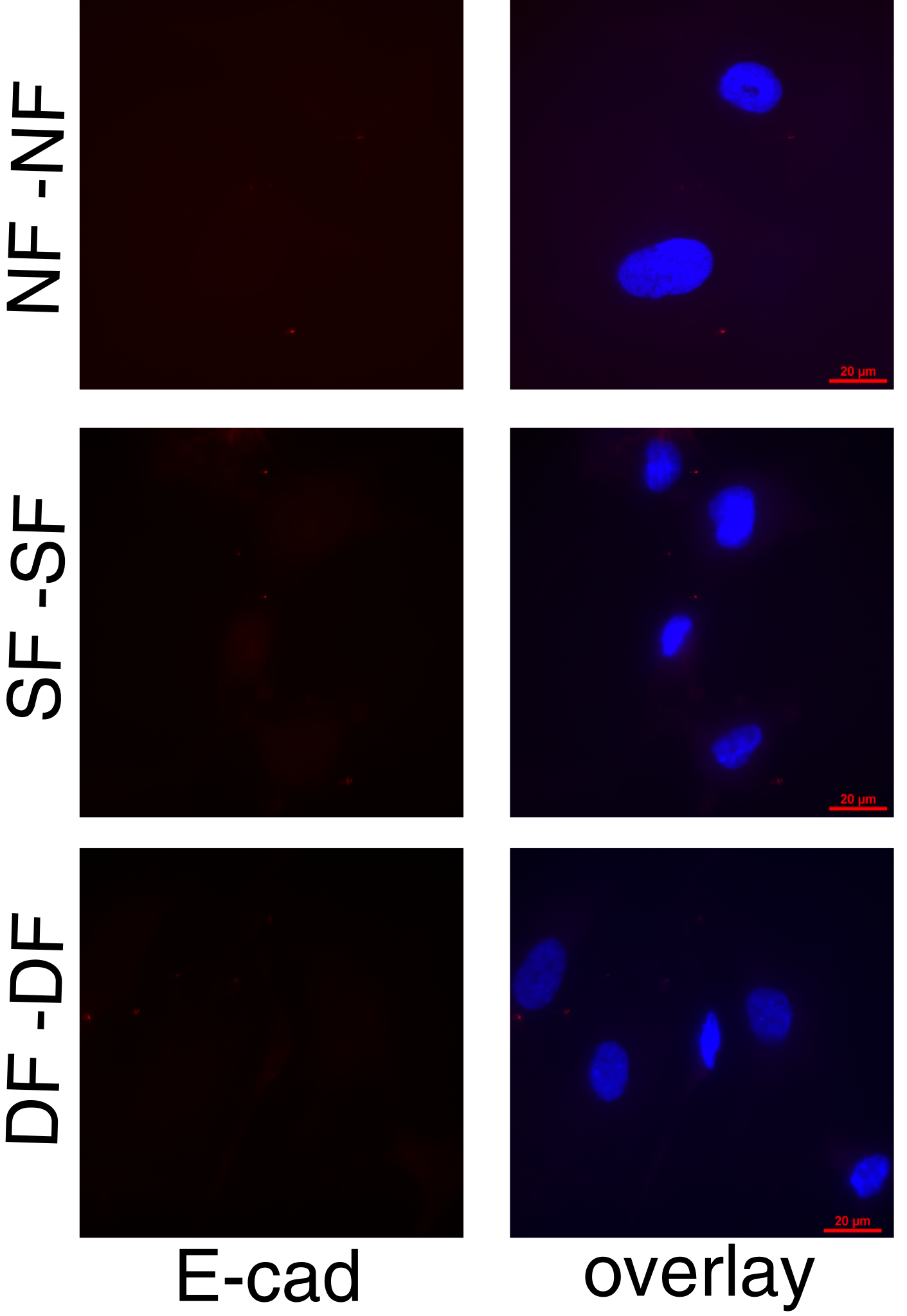
**

**Supplementary figure 3** Immunostaining of fibroblasts adherens junctions for E-cadherin shows no expression of E-cad in the NF-NF, SF-SF and DF-DF interaction sites. Scale bar 20 µm.

**
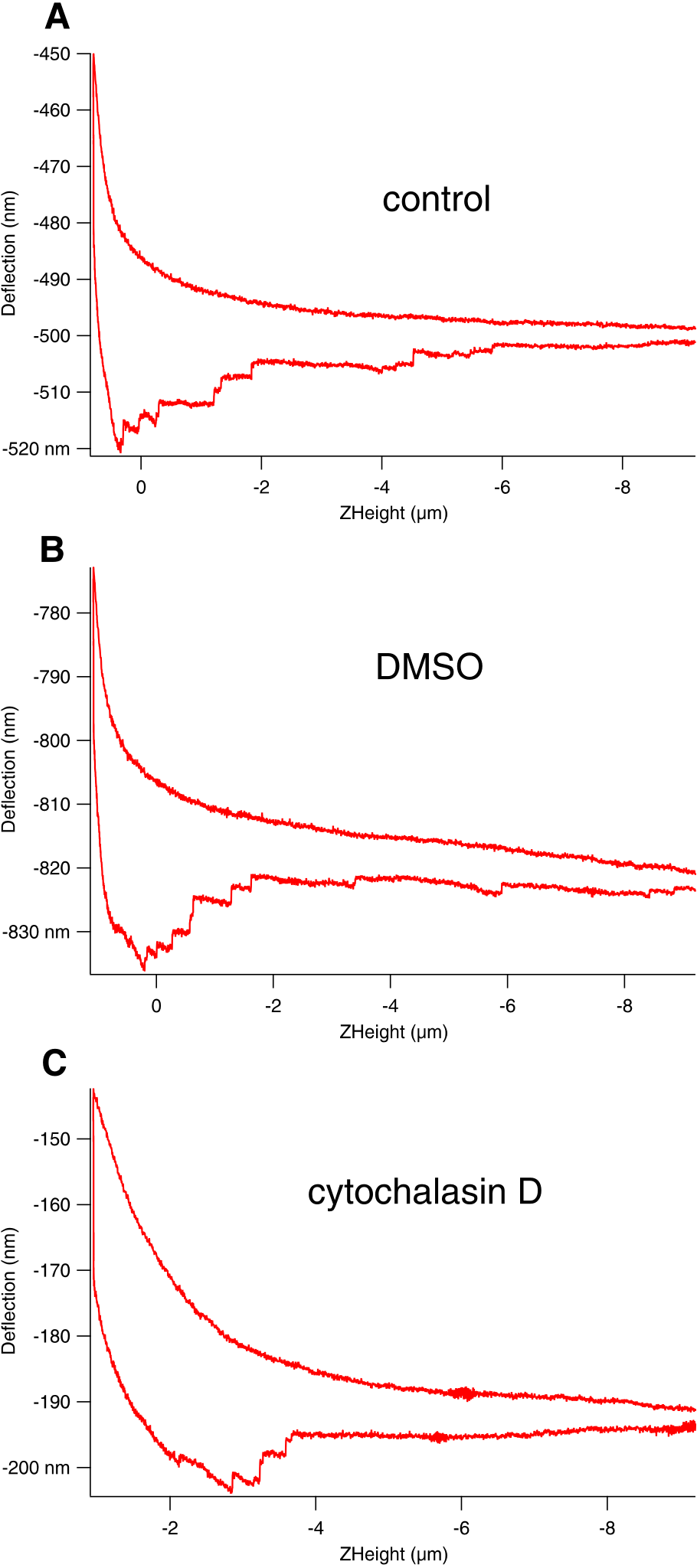
**

**Supplementary figure 4** Force curves measured during cell-cell interaction (here for NF-NF) shows rupture events in all three experimental conditions: control in normal DMEM medium (A), medium plus DMSO (B), and medium with cytochalasin D (C).

**
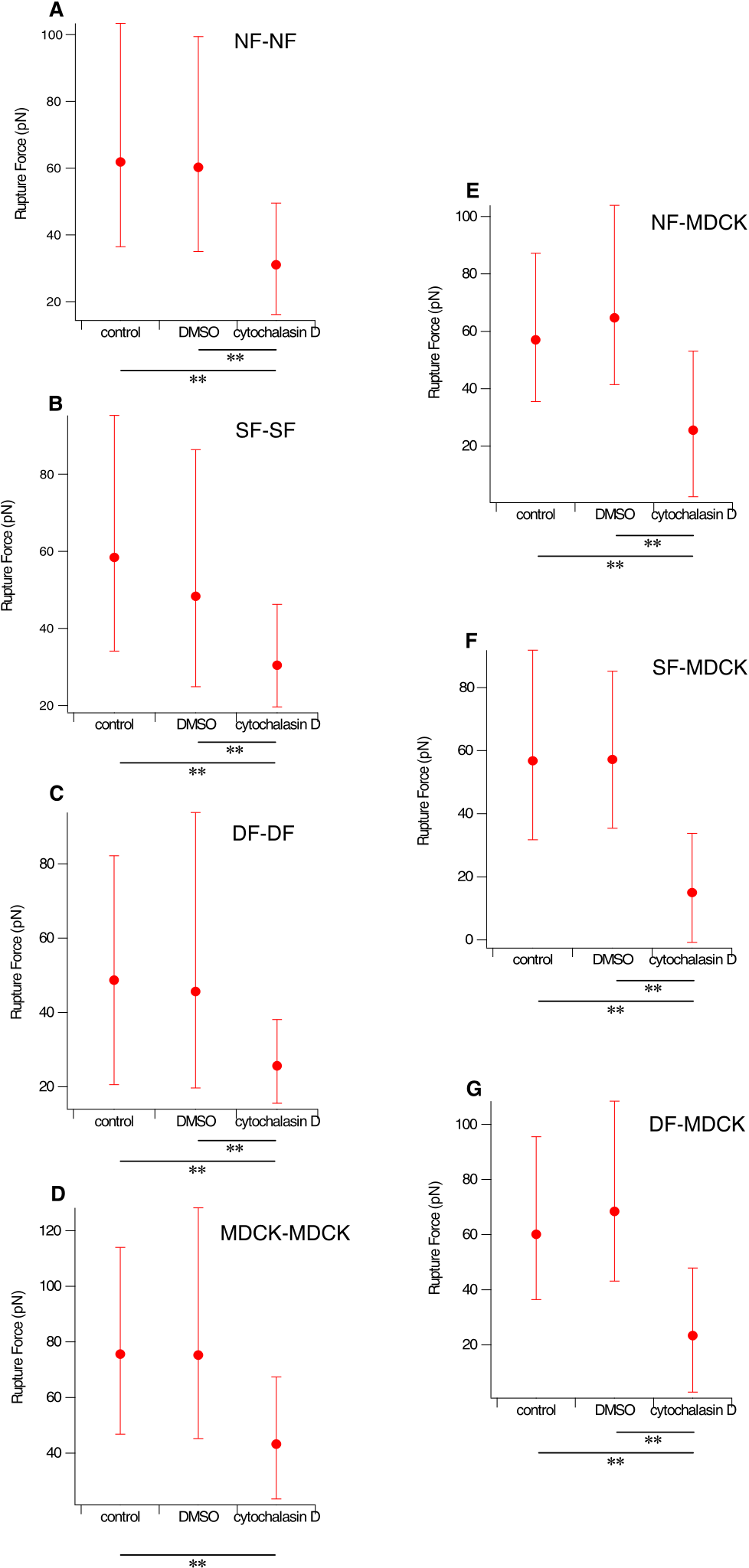
**

**Supplementary figure 5** Plot of the median values with the 25th and 75th percentile added as error bars of rupture forces in cell-cell interactions between several cells under control, DMSO and cytochalasin D (5 µM) conditions. (A) NF-NF, (B) SF-SF, (C) DF-DF, (D) MDCK-MDCK, (E) NF-MDCK, (F) SF-MDCK and (G) DF-MDCK. The respective median values are also listed in Table 2. n=2 for each category; n represents the number of independent experiments performed for each category (see Materials and Methods section). Statistical results are reported in Materials and Methods section.

**
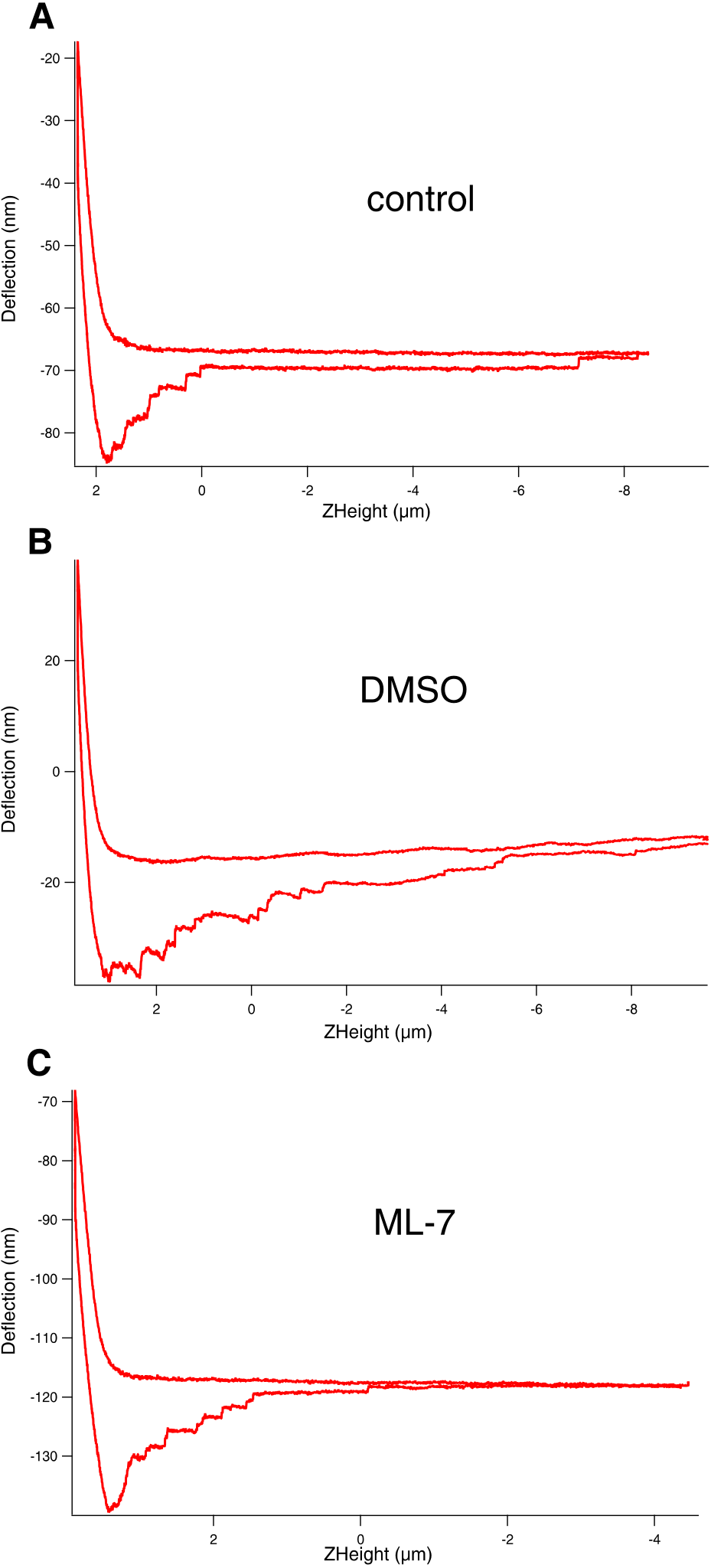
**

**Supplementary figure 6** Force curves measured during cell-cell interaction of normal fibroblasts (NF-NF) shows rupture events in all three experimental conditions: control in normal medium (A), medium plus DMSO (B), and medium with ML-7 (C).

**
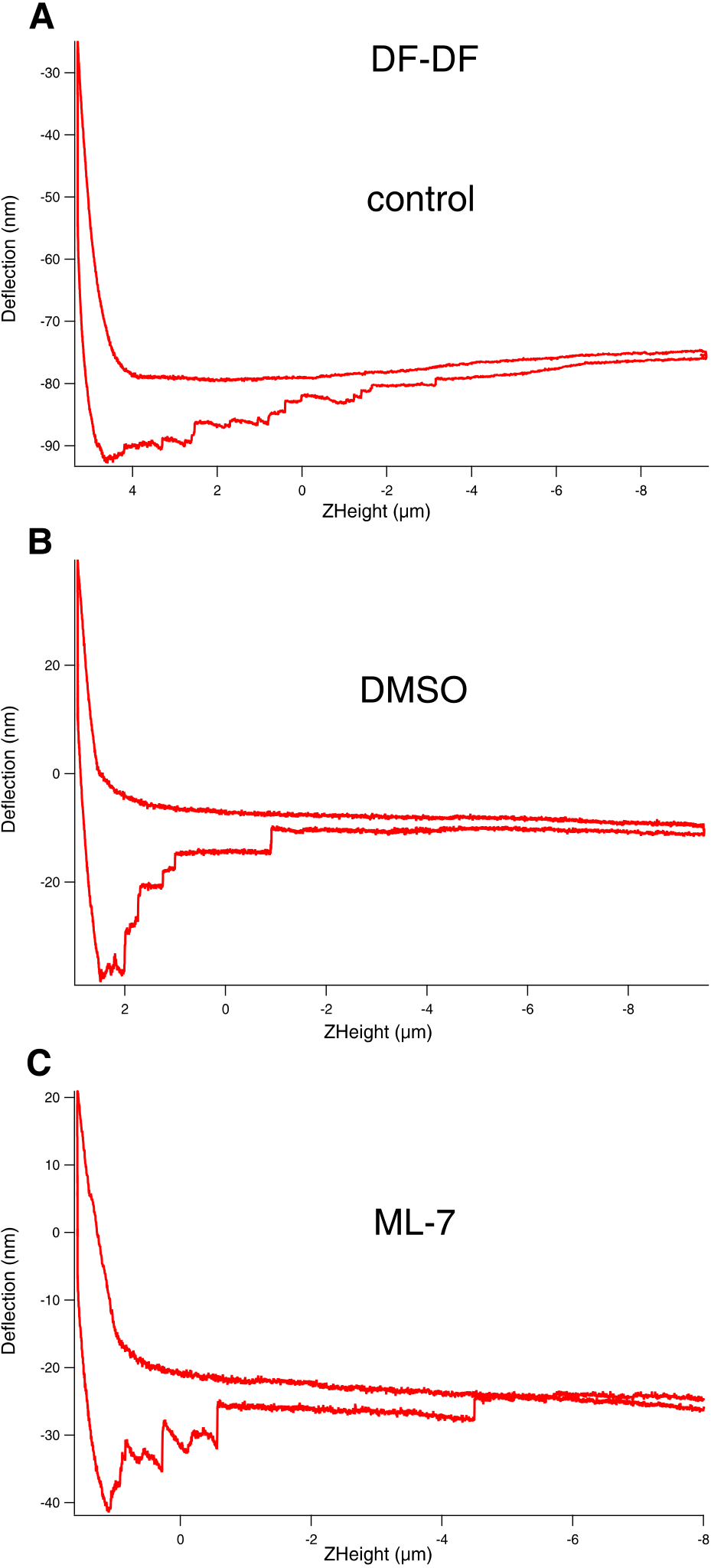
**

**Supplementary figure 7** Force curves measured during cell-cell interaction of Dupuytren fibroblasts (DF-DF) shows rupture events in all three experimental conditions: control in normal medium (A), medium plus DMSO (B), and medium with ML-7 (C).


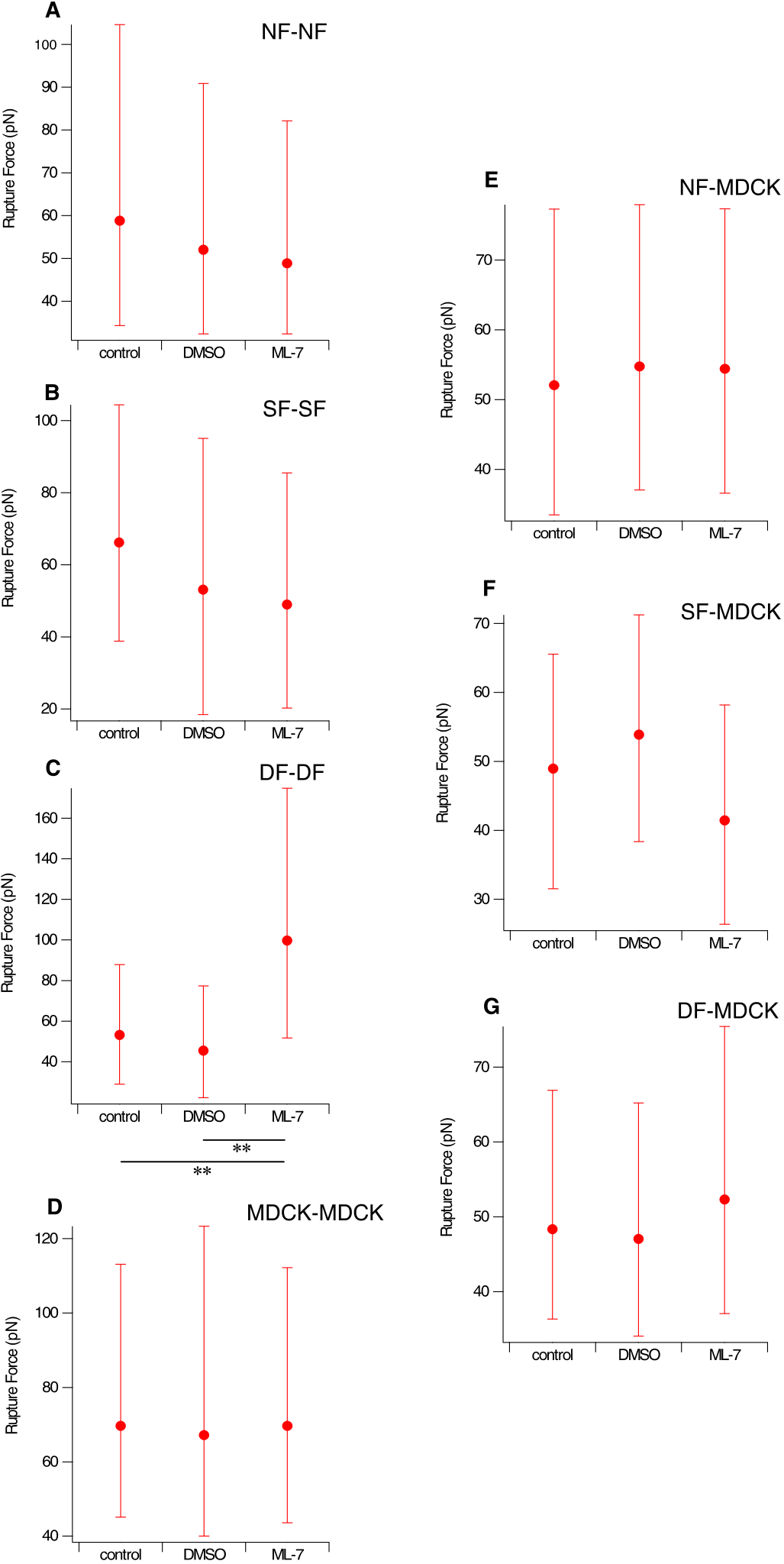


**Supplementary figure 8** Plot of the median values with the 25th and 75th percentile added as error bars of rupture forces in cell-cell interactions between several cells under control, DMSO and ML-7 (5 µM) conditions. (A) NF-NF, (B) SF-SF, (C) DF-DF, (D) MDCK-MDCK, (E) NF-MDCK, (F) SF-MDCK and (G) DF-MDCK. The respective median values are also listed in Table 3. n=2 for each category; n represents the number of independent experiments performed for each category (see Materials and Methods section). Statistical results are reported in Materials and Methods section.
